# Integrating Dense Array EEG in the Presurgical Evaluation of Temporal Lobe Epilepsy

**DOI:** 10.5402/2012/924081

**Published:** 2012-11-14

**Authors:** Madoka Yamazaki, Marie Terrill, Ayataka Fujimoto, Takamichi Yamamoto, Don M. Tucker

**Affiliations:** ^1^Comprehensive Epilepsy Center, Seirei Hamamatsu General Hospital, 2-12-12 Sumiyoshi, Naka-ku, Hamamatsu, Shizuoka 4308558, Japan; ^2^Electrical Geodesics, Inc., Eugene, OR 97403, USA; ^3^Department of Psychology, University of Oregon, Eugene, OR 97403, USA

## Abstract

*Purpose*. To evaluate the clinical utility of dense array electroencephalography (dEEG) for detecting and localizing interictal spikes in temporal lobe epilepsy. *Methods*. Simultaneous invasive and noninvasive recordings were performed across two different groups. (1) The first group underwent both noninvasive recording with 128 channels of (scalp) dEEG and invasive sphenoidal electrode recording. (2) The second group underwent both noninvasive recording with 256 channels of (scalp) dEEG and invasive intracranial EEG (icEEG) involving coverage with grids and strips over the lateral and mesial temporal lobe. A noninvasive to noninvasive comparison was made comparing the overall spike detection rate of the dEEG to that of conventional 10/20 EEG. A noninvasive to invasive comparison was made comparing the spike detection rate of dEEG to that of conventional 10/20 EEG plus sphenoidal electrodes. And finally, a noninvasive to invasive evaluation measuring the source localization ability of the dEEG using the icEEG as validation. *Results*. In the 128-channel dEEG study (1), 90.4% of the interictal spikes detected by the dEEG were not detected in the 10/20 montage. 91% of the dEEG-detected spikes were accurately localized to the medial temporal lobe. In the 256-channel dEEG study (2), 218 of 519 interictal spikes (42%) were detected by dEEG. 85% of these spikes were accurately localized to the medial temporal lobe, close to the position confirmed by subdural electrodes. *Conclusion*. Dense array EEG may provide more precise information than conventional EEG and has a potential for providing an alternative to sphenoidal electrode monitoring in patients with temporal lobe epilepsy.

## 1. Introduction

Epilepsy is a disorder of recurrent seizures affecting up to 1% of the world's population [1, 2]. Temporal lobe epilepsy (TLE) is the most prevalent and difficult to treat form of the disorder (9), and it is the most common cause of pharmacoresistant seizures [3–5]. Approximately 30% of epileptic patients continue to have seizures after treatment with antiepileptic drugs (AEDs) [6]. After several drug trials and combinations, if patients either cannot tolerate the drug's side effects or continue to have uncontrolled seizures, they may be referred for surgical evaluation. Outcome studies find that 50%–80% of patients with temporal lobe epilepsy can expect to become seizure-free following surgery [7–10]. To be determined to be a viable surgical candidate, a patient's seizures must be determined to have a focal zone of onset that can be targeted for surgical resection.

Typically, presurgical evaluation involves conventional LTM EEG recording with 19 scalp electrodes. In cases of TLE, sphenoidal electrodes may be used to more fully assess discharges from temporal regions. While the use of sphenoidal electrodes has been shown to provide additional unique localizing information of seizure activity, the overall added positive data yield is actually quite low, only about 5%–10% [11]. Recently, it has become possible to record dense array EEG (dEEG) with 128 or 256 channels in the clinical setting. In addition to sampling epileptic discharges from the whole head surface, dEEG allows electrical source imaging (ESI) of the neural generators, through inverse estimation with a computational model of the conductivity of head tissues [12–15]. Although dEEG localization of seizure onset is preferred [16], dEEG localization of spikes alone has been shown to be more effective in predicting the seizure onset zone than other methods including PET, MRI, and ictal SPECT.

In the present study, we evaluated the ability of dEEG compared with conventional EEG to detect interictal epileptiform spikes at the scalp generated by the mesial temporal lobe across two different groups of patients. Additionally, in the group that were identified as surgical candidates, the ability of 256-channel dEEG to reasonably localize the source of the epileptiform activity was evaluated.

One group consisted of three patients with TLE who were being evaluated for surgical candidacy. These patients underwent 128-channel dEEG with simultaneous sphenoidal electrode recording. The spike detection ability of the 128-channel dEEG was compared to the spike detection ability of the 19-channel subset of the 10/20 equivalent electrodes within the net. Additionally, the spike detection ability of the dEEG was compared to the spike detection ability of the 10/20 equivalent electrodes plus the data from the sphenoidal electrodes, which are often used to detect activity in temporal lobe epilepsy that are outside of the area covered by the 10/20 electrode placement system.

The second group consisted of three patients, also with TLE, who had already been identified as surgical candidates. These patients underwent 256-channel dEEG with simultaneous intracranial EEG (icEEG) recording. Similar to the first group, the spike detection ability of the dEEG to detect interictal spikes from the mesial temporal lobe was compared to the spike detection ability of the 10/20 equivalent electrodes. In addition for this group, the ability of the dEEG to reasonably localize the source of the interictal activity was evaluated using the icEEG as a standard of validation.

## 2. Methods

### 2.1. Patients

We received approval for this study from Seirei Hamamatsu General Hospital Human Subject Committee and informed consent was obtained from all patients.

We studied six patients who were selected for this study based on the criteria that they were diagnosed with mesial temporal lobe epilepsy (mTLE) based on seizure semiology, EEG and positive MRI findings and had suffered medically refractory mTLE for at least 2 years.

Each patient underwent a presurgical workup including conventional LTM EEG monitoring, MRI structural imaging, 125-Iomazenil (IMZ)-singlephoton emission tomography (SPECT) and neuropsychological testing. The clinical information for these patients is summarized in Table 1.

### 2.2. Dense Array EEG Recording

#### 2.2.1. Simultaneous 128-Channel dEEG and Sphenoidal Electrode Recording

**Figure 1:**
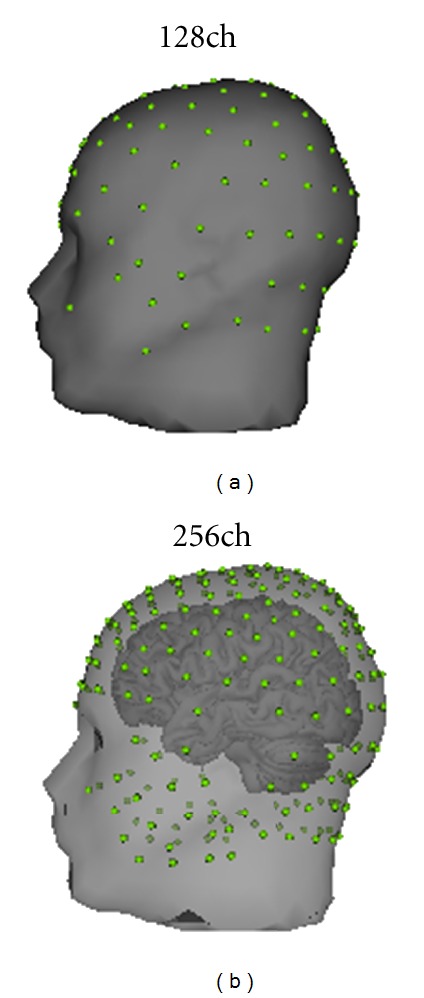
(a) 128-channel dEEG electrodes position. (b) 256-channel dEEG electrodes position.

To determine surgical candidacy, patients 1, 2 and 3 underwent LTM EEG with the 128 channel Geodesic Sensor Net (Electrical Geodesics Inc., Eugene OR, USA) and simultaneous sphenoidal electrode recording (NicoletOne CareFusion, Middleton WI, USA). The AgCl electrodes of the dense array net are interconnected in a geodesic structure and are spaced with approximately 3 cm interelectrode distance, thus providing evenly spaced electrode coverage of the whole head. The net was adjusted so that electrodes over the Vertex, Nasion, Inion and pre-auricular points were correctly located according to the international 10/20 system. The layout of the sensor array is shown in Figure 1(a). The dEEG data was collected using the Net Amps 300, a high-input impedance amplifier (Electrical Geodesics Inc., Eugene OR, USA) with a 500 Hz sampling rate and 0.1–200 Hz bandpass filter.

Sphenoidal electrodes were inserted from just below the inferior margin of the zygomatic arch and between the coronoid and condylar processes of the mandible under local anesthesia by a trained neurosurgeon.

For this patient group, spikes detected by the sphenoidal electrodes were used as a validation method to determine whether interictal spikes generated by the temporal lobe propagated to the scalp surface and whether the dEEG could better detect the interictal spikes.

Additionally, sphenoidal electrode data that served as a validation method of dEEG's ability to estimate the source of the measured scalp activity and calculate it back to the temporal lobe was evaluated. 

It is important to note that after presurgical evaluation, the patients in this group were identified as surgical candidates, however they elected to not undergo resective surgery.

#### 2.2.2. Simultaneous 256-Channel dEEG and icEEG Recording

At the time of this study, patients 4, 5, and 6 had already been identified as candidates for epilepsy resective surgery and were in the icEEG (phase II) stage of evaluation. These patients underwent 256-channel dEEG with the Net Amps 300 (Electrical Geodesics Inc., Eugene OR, USA) and simultaneous icEEG (NicoletOne CareFusion, Middleton WI, USA) recordings.

Subdural electrodes were placed in order to delineate the epileptogenic zone for cortical excision and to separate it from functional areas. Each patient had from 56 to 64 subdural strip and grid electrodes implanted over the mesial and lateral temporal lobe. All contacts were platinum, and the interelectrode distance was 5 mm for the T-shaped electrodes and 10 mm for the other electrodes.

By day 3 following the icEEG implantation, the scalp wound had healed sufficiently to allow simultaneous dEEG recording with minimal infection risk. The 256-channel Geodesic Sensor Net also distributes standard AgCl electrodes in a geodesic structure, with a typical interelectrode distance of 20–25 mm. The net was adjusted so that electrodes over the vertex, nasion, inion, and preauricular points were correctly located according to the international 10/20 system. The layout of the sensor array is shown in Figure 1(b).

Both the dEEG and the icEEG were recorded at 1000 Hz sampling rate with a 0.1–400 Hz bandpass filter. Simultaneous data was collected for 30–40 minutes with no complications.

For this patient group, the icEEG served as a validation method for the simultaneously recorded dEEG. For example, the icEEG could be used to determine whether interictal spikes generated by the mesial temporal lobe propagated to the scalp surface and whether the dEEG could better detect the interictal spikes. Additionally, the ability of the dEEG to estimate the source of the measured scalp activity and calculate it back to the mesial temporal lobe was evaluated.

#### 2.2.3. EEG Data Synchronization

A digital pulse from the sphenoidal electrodes and icEEG recording system was provided to the dEEG acquisition system for synchronization.

### 2.3. Data Analysis

For the simultaneous 128-channel dEEG and sphenoidal electrode recordings, we manually selected interictal spikes from 30–40 minute periods of artifact-free data. The maximum amplitude of each spike was calculated using the full 128-channel data set and compared against which electrode showed maximum amplitude in the subset of just the conventional 10/20 equivalent electrodes viewed in a standard chart montage.

For the simultaneous 256-channel dEEG and icEEG recordings, frequent interictal spikes were marked during artifact-free periods from the dEEG and mesial temporal lobe spikes in the icEEG were marked by visual inspection. For each icEEG spike, we evaluated whether that same spike propagated to and was detected on the scalp by both the dEEG data and the conventional set 10/20 electrode subset.

Electrical source localization was conducted at the rising phase for each of the spikes recorded by the dEEG (128 or 256) using the Geosource 1.0 source localization software package (http://www.egi.com/). Source calculations were performed within the space of a 3D head model derived from the Montreal Neurological Institute's average adult MRI and using the linear inverse method LAURA (local autoregressive average) [12, 13]. The LAURA constraint provides results very similar to the LORETA (spatial laplacian) constraint [17] and has been shown to provide a stable source estimation of interictal epileptiform events in neurosurgical planning for epilepsy [18–20].

## 3. Results

### 3.1. Simultaneous 128-Channel dEEG and Sphenoidal Electrodes Recording

**Figure 2:**
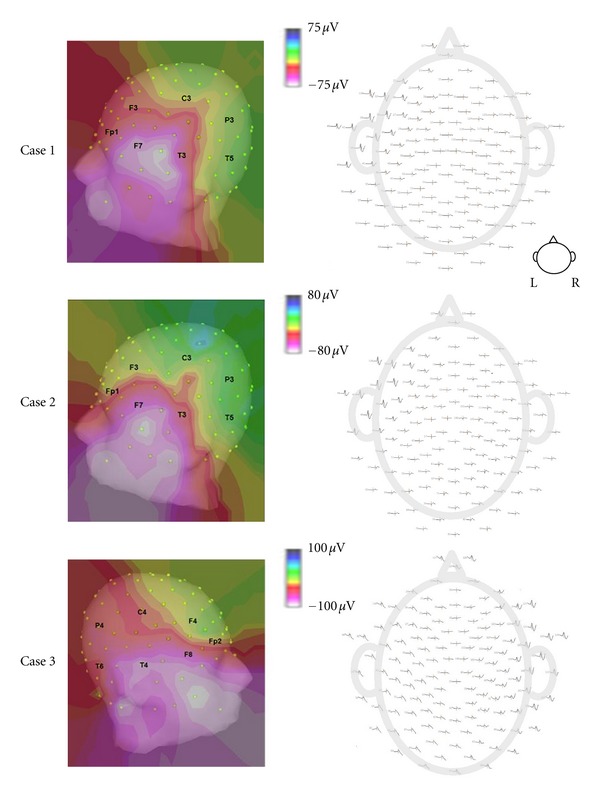
Spike voltage topography. (Left) Averaged spike voltage topography; white color shows maximal spike amplitude. (Right) 128-channel dEEG topoplot.

A total of 104 spikes was captured by the dEEG across three patients (cases 1–3). All spikes recorded by the sphenoidal electrodes were also detected by the dEEG. All 104 spikes were located in the anterior or basal temporal lobe regions. The averaged spike voltage topography for each patient is shown in Figure 2. With data only from the conventional 10/20 electrode equivalents, we found that 10 (9.6%) of the 104 total spikes were detected. The 10 spikes detected in the 10/20 equivalent electrodes had a maximum amplitude at either electrode F7 or F8, which are the two electrodes from the conventional 10/20 array to cover the anterior temporal areas.

The other 90.4% of the spikes detected by the dEEG were outside of the 10/20 electrode array and therefore were not detected in the 10/20 montage.

**Figure 3:**
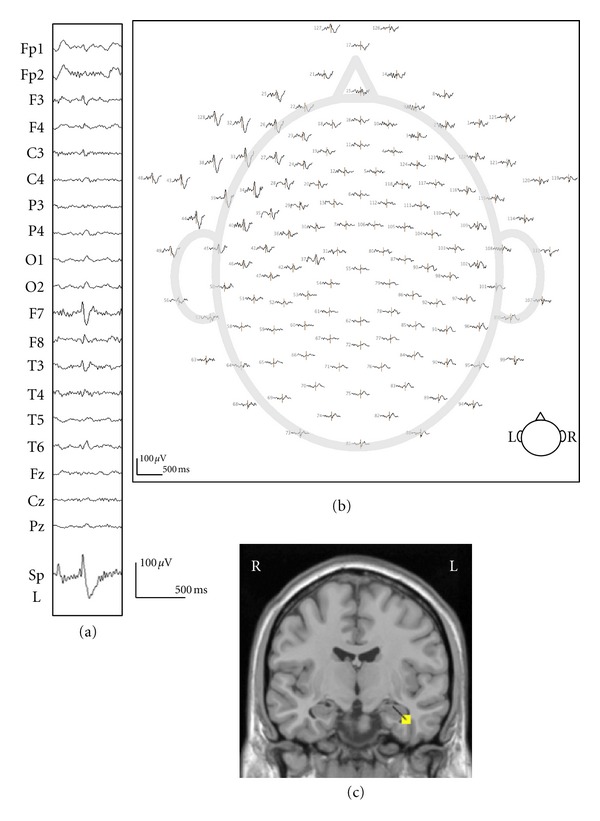
A typical example of simultaneous recording 128-channel dEEG and sphenoidal electrode (case 1). (a) The EEG (upper) shows a left temporal spike in 19-channel 10/20 display. The lower shows simultaneously recorded left side of sphenoidal EEG. (b) 128-channel dEEG topographic plot of the corresponding spike. The view is looking down on top of the head with nose at the top. The distribution of spike discharge is over the left anterior temporal electrodes. (c) The source estimation by dEEG superimposed on a standard MRI. The interictal spike is localized to left mesial temporal region.

A typical example of the Geosource source estimation results from a dEEG and sphenoidal electrode recording is shown in Figure 3. All spikes had source estimations located in the temporal lobe. Out of the 104 spikes, 95 were well localized in the mesial temporal lobe, as validated by the sphenoidal electrode recordings, which is equivalent to 91% accuracy.

### 3.2. Simultaneous 256-Channel dEEG and ic EEG Recording

During the 30–40 minutes of simultaneous dEEG and icEEG recording, a total of 519 icEEG spikes were recorded from mesial temporal regions (cases 4–6). Of the 519 spikes recorded by the icEEG, 218 of these spikes (42%) were also clearly distinguishable from background activity in the 256 channel dEEG. The dEEG detection rate of icEEG spikes for each patient was 42% in Case 4, 38% in Case 5, and 47% in Case 6.

**Figure 4:**
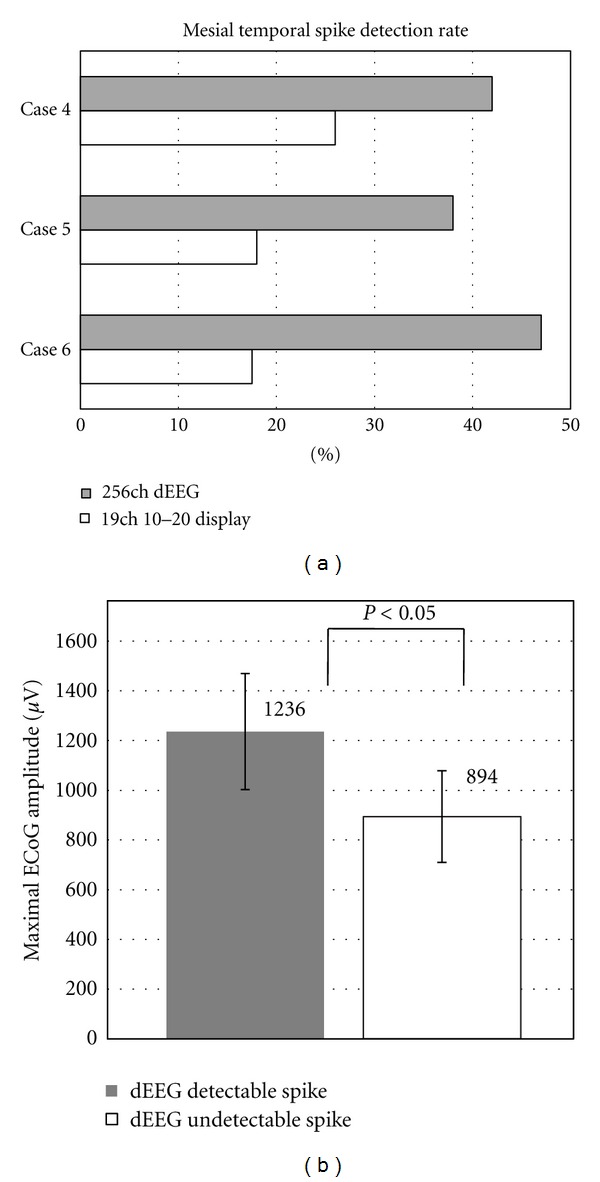
(a) Spike detection rate for 256-channel dEEG and 19-channel 10/20 display. (b) Maximal amplitude of icEEG.

Comparatively, when the data was spatially down sampled to just the conventional 10/20 electrode equivalents, the spike detection rate decreased to 26% in Case 4, 18% in Case 5, and 17% in Case 6 (Figure 4(a)).

The average maximum amplitude of the spikes that were detected by both the icEEG and the dEEG data was 1236 *μ*V (standard deviation of 233 *μ*V). This is significantly higher (*P* < 0.05) than the average maximum amplitude of the spikes that were detected by the icEEG data but not detected at the scalp by the dEEG (894 *μ*V with standard deviation of 184 *μ*V) (Figure 4(b)).

**Figure 5:**
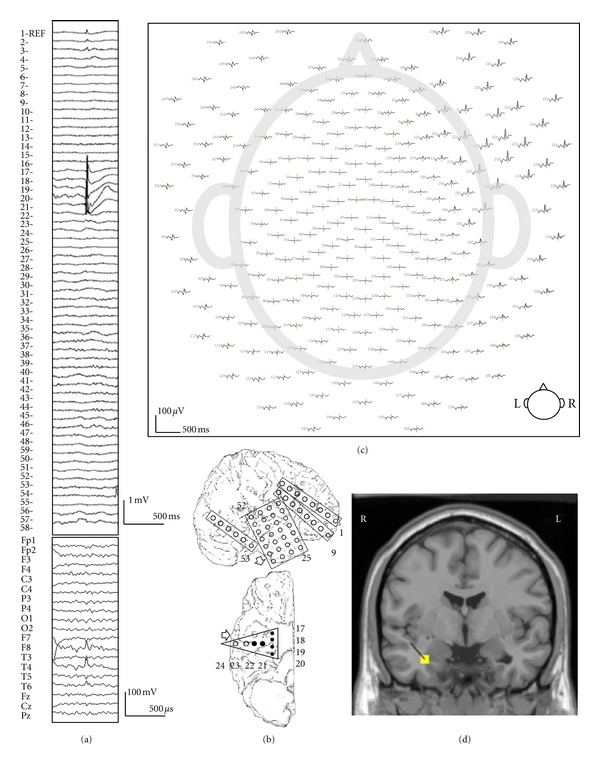
A typical example of 10/20 display undetectable spike (case 5). (a) The icEEG (upper) shows a right mesial temporal spike. Interictal spike is shown at electrodes nos. 17–22 which are located over the mesial temporal region. The EEG (lower) simultaneously recorded 256-channel dEEG with 19-channel 10/20 display. (b) Placement of subdural electrodes and the location of the interictal spike. Solid circle indicates the electrodes which show the interictal spike. (c) 256-channel dEEG topographic plot of the corresponding spike. The view is looking down on top of the head with nose at the top. The distribution of spike discharge not seen in the 10/20 montage is over the right-face electrodes (right upper corner). The 256-channnel topographic plot was instructive in localizing the spike to the anterior basal surface of the temporal lobe. (d) The source estimation by dEEG is superimposed on a standard MRI. The interictal spike is localized to the right mesial temporal region.

When source estimations were performed on each of the spikes detected in the dEEG, all of them were correctly localized to the temporal lobe. 185 of the 218 spikes (85%) were well localized in the mesial temporal lobe, close to the position confirmed by the subdural electrodes. A typical example of the 256 dEEG source estimation and the corresponding icEEG is shown in Figure 5.

Based on the intracranial EEG findings, all three patients in this group underwent anterior temporal lobectomy with amygdalohippocampectomy. The surgical outcome for each is Engel class I within the postoperative follow up period of 11 to 27 months (mean of 24 months) at the time this publication was written.

## 4. Discussion

Of the 104 interictal spikes captured in the 128-channel recordings, only 10 were detected by the conventional 10/20 electrodes. For the other 94, the maximum amplitude of these spikes was detected in sensors on the face and neck, outside the conventional 10/20 electrode array. In order to measure spikes from basal and anterior temporal cortex, it is common to use sphenoidal electrodes in addition to the 10/20 electrode array for temporal lobe epilepsy patients [20]. Although many studies have shown the utility of sphenoidal electrodes [11, 21–26], several studies have shown that scalp electrodes placed in the anterior temporal regions can capture as much or more than the sphenoidal electrodes [27–32].

In the present study, all spikes recorded by the sphenoidal electrodes were also detected by the 128-channel dEEG, suggesting that dEEG could provide equivalent information while eliminating the pain and risk of sphenoidal electrodes.

The 256-channel sensor net provides not only greater sensor density but also improved coverage of the face and neck. For the three patients examined with 256-channel dEEG, the simultaneous icEEG provided a validation method for evaluating temporal lobe spikes detected at the scalp surface that were also seen with invasive monitoring. Of the spikes detected with icEEG, only 42% were detected with the 256-channel dEEG on average in these patients, a rate consistent with previous simultaneous icEEG and dEEG studies [14]. Also consistent with the previous findings, the spikes that were detected with dEEG were larger than those not detected. Such results imply that there are many smaller spikes detectable with icEEG that are not seen through typical visual inspection of the noninvasive dEEG recordings, even with 256 channels.

Even the smaller spikes, of course, are volume-conducted to the head surface and are, at least theoretically, detectable in the dEEG signals. An interesting challenge for future research is whether more sensitive signal processing could improve the detection of small spikes and other epileptiform events (such as high frequency oscillations) compared to the visual inspection used in the present study.

A comparison of the spike detection results from 256-channel dEEG with those from conventional (international ten-twenty positions) EEG was obtained by downsampling the 256 array to 19 channels. The detection of icEEG spikes dropped from 42% to 26% in the first patient, from 38% to 18% in the second, and from 47% to 17% in the third. Clearly, the yield from noninvasive EEG is improved through the use of the dense array.

An important question is whether the improvement in detecting spikes with dEEG versus conventional EEG is clinically significant. A similar question could be asked in comparing the dEEG detection with that from icEEG. If the same epileptic tissue is generating the small spikes as that generating large spikes, then perhaps only detecting large spikes may be sufficient to determine the likely seizure onset zone.

Certainly, the localization of spikes with dEEG is superior to that with conventional sparse array EEG. Of the 218 spikes detected with the 256-channel array in the present patients, 85% were well localized to the correct location as confirmed by the icEEG. As shown in the recent clinical trial in Geneva [32], accurate localization of the patient's typical spike localization can be clinically significant in guiding neurosurgical resection of the seizure onset zone.

For many years, it was thought that localization of interictal sources with magnetoencephalography (MEG) is superior to that with EEG, primarily because the magnetic field of neural sources is not distorted by the resistive tissues of the head, particularly the skull. Recent measurement of the electrical resistivity of the human skull has shown that it is not as resistive as formerly thought. Measurement in vitro and estimation in vivo suggested a 14 : 1 skull to brain resistivity ratio versus the previously assumed values of 80 : 1 [33]. A less resistive skull means that the source localization accuracy with EEG is roughly comparable to that obtained with MEG, with the accuracy of both depending on sensor density [34]. Furthermore, MEG is relatively insensitive to radially oriented sources, and these are common for tissues generating spikes in the medial temporal lobe.

There have been a few studies obtaining simultaneous recordings of epileptic spikes with whole head MEG and icEEG, allowing comparison of spike detection rates with the present dEEG and icEEG recordings. Mikuni et al. [35] reported that MEG detected 18% of medial temporal spikes that were detected by simultaneous icEEG. Oishi et al. [36] reported that MEG detected only 26% of medial temporal spikes seen by icEEG but detected 53% of lateral frontal spikes, perhaps consistent with the greater radial orientation (and less MEG sensitivity) of medial temporal versus lateral cortical sites. More recently, Huiskamp et al. [37] reported that only 28% of medial temporal lobe spikes confirmed by icEEG were detected by MEG, whereas the MEG detection increased to 70% for spikes from lateral cortical sites.

The medial temporal spike detection rates for MEG in these studies (18%, 26%, and 28%) are comparable to the rates for the conventional 19-channel EEG montage in the present study (26%, 18%, and 17% in the three patients) and are considerably poorer than the detection rate with 256 dEEG (42%, 38%, and 47%, resp.).

Adequate spatial sampling of the scalp surface with dEEG thus increases spike detection sensitivity, and it also increases the accuracy of source localization in presurgical epilepsy evaluation. In contrast with MEG, 256-channel dEEG can be implemented for long term monitoring for detecting and localizing seizure onset [16]. Integrating dEEG in the protocol for presurgical evaluation may therefore improve the patient's chances for being selected as a successful surgery candidate, and it may decrease the uncertainty in the placement of the icEEG electrodes. For the patients with 256-channel dEEG in the present study, all three underwent anterior temporal lobectomy with amygdalohippocampectomy. The surgical outcome for each is Engel class I within the postoperative followup period of 11 to 27 months (mean of 24 months) at the time of this writing.

## 5. Conclusion

This study shows the clinical usefulness of dEEG in the presurgical evaluation of mTLE. Specifically, as validated by the sphenoidal and icEEG data, dEEG has increased ability to both detect interictal spikes at the scalp compared to conventional 10/20 EEG and also increased ability to calculate the electrical source of the interictal activity with reasonable accuracy.

Therefore, dense array EEG may provide more precise information than conventional EEG and can potentially provide an alternative to sphenoidal electrode monitoring in patients with temporal lobe epilepsy, making the presurgical evaluation less invasive.

## Figures and Tables

**Table 1:** 

	Case 1	Case 2	Case 3	Case 4	Case 5	Case 6
Age/sex	34 y.o/F	42 y.o/M	34 y.o/M	18 y.o/M	36 y.o/M	16 y.o/F
Sz onset	10 y.o	9 y.o	10 y.o	13 y.o	4 y.o	12 y.o
Sz type	SPS, CPS	CPS	SPS, CPS	SPS, CPS, sGTC	CPS, sGTC	SPS, CPS, sGTC
MRI	L HA	L HA	R mT, P cortical displasia	R amygdala tumor	R HA	L HA
IMZ-SPECT*	L mT	—	R mT, laT, P	R mT	R mT, laT	L mT, laT

SPS: simple partial seizure, CPS: complex partial seizure, sGTC: secondary generalized tonic clonic seizure.

*Hypoperfusion area.

—: not examined.

mT: mesial temporal lobe, laT: lateral temporal lobe, P: parietal lobe.
